# Construction of indicator system and comprehensive evaluation of high-quality development of cultural tourism integration in revolutionary base areas

**DOI:** 10.1371/journal.pone.0347333

**Published:** 2026-05-05

**Authors:** Ping Li, Chunxiao Xu, Huiting Liang

**Affiliations:** School of Tourism, Hunan Normal University, Changsha, Hunan, China; Macau University of Science and Technology, MACAO

## Abstract

Clarifying the conceptual framework of high-quality development in cultural-tourism integration and establishing a corresponding evaluation system suitable for county-level regions in revolutionary base areas are crucial for revitalizing these regions in the new era. Guided by national strategies, informed by local practices, and tailored to the regional characteristics of revolutionary base areas, this study employs a mixed-method approach combining grounded theory and expert consultation to develop a scientifically robust evaluation indicator system for county-level high-quality development in cultural-tourism integration. Using the Former Central Soviet Area of Jiangxi-Fujian-Guangdong as a case study, the research reveals three key findings: 1) The core connotation of high-quality development in cultural-tourism integration in revolutionary base areas centers on leveraging tourism activities rooted in red culture to drive cross-sector industrial integration, thereby enhancing the level, quality, and sustainability of economic and social development. 2) The evaluation indicator system comprises 21 indicators organized into three dimensions: development elements, development effects, and development environment. 3) The high-quality development level of cultural-tourism integration in the Former Central Soviet Area demonstrates a fluctuating upward trend over time. Spatially, low-level areas show contraction while high-level areas expand, accompanied by a gradually intensifying trend of spatial agglomeration.

## Introduction

High-quality development represents the central mission in China’s endeavor to comprehensively build a modern socialist country. Since the initiation of reform and opening-up, the implementation of an imbalanced regional development strategy has led to the formation of a “core-periphery” spatial structure in China, mirroring patterns observed globally [1]. Reducing regional disparities and promoting coordinated development have thus emerged as an international consensus [2,3]. The 19th National Congress of the Communist Party of China explicitly called for advancing high-quality development and constructing a new development paradigm, integrating the regional coordinated development strategy with the revitalization of special-type areas [4,5].

As a typical category of such areas, revolutionary base areas span 1,389 counties (cities, districts) across 28 provinces (autonomous regions, municipalities) and endured heavy sacrifices during the Agrarian Revolutionary War and the Anti-Japanese War [6]. However, constrained by historical factors such as environmental limitations and war-induced trauma, these areas have long suffered from socio-economic stagnation [7], constituting a major bottleneck in China’s coordinated regional development [8]. In 2021, the State Council successively issued the *Opinions on Supporting the Revitalization and Development of Revolutionary Base Areas in the New Era* and the *14th Five-Year Plan for the Revitalization of Special-Type Regions*, clearly outlining objectives for promoting high-quality development in revolutionary base areas. The report of the 20th CPC National Congress further proposed to “enrich tourism through culture and boost culture through tourism, advancing the deep integration of culture and tourism,” thereby establishing cultural-tourism integration as a strategic priority for achieving high-quality development. Additionally, the *2022 Plan to Promote High-Quality Development of Red Tourism in Revolutionary Base Areas* highlighted the pivotal role of integrated cultural and tourism development in driving regional revitalization.

Against this backdrop, leveraging cultural-tourism integration to optimize resource allocation, drive industrial innovation and product upgrading, and foster cross-regional complementarity and collaborative clustering has emerged as a crucial pathway to stimulate consumption upgrades, achieve holistic socio-economic progress, and ensure sustainable development. Deconstructing the conceptual framework of high-quality development in cultural-tourism integration and constructing a customized evaluation indicator system for county-level revolutionary base areas carry substantial practical relevance for preserving the legacy of red culture and realizing comprehensive revitalization in the new era. Such a system can offer theoretical guidance for cultural-tourism integration practices in these regions while enabling targeted interventions through quantitative assessment to address development disparities, thereby advancing the goal of common prosperity.

Globally, developed countries have put forward various concepts and indicator systems analogous to high-quality development. Examples include the State New Economy Index Report of the United States [9], the EU’s Europe 2020 Strategy [10], South Korea’s Green Growth Strategy [11], and Japan’s New Growth Strategy [12]. While these nations differ in their developmental contexts, their indicator systems consistently emphasize innovation-driven growth, industrial transformation and upgrading, ecological conservation, and improvements in people’s livelihoods, reflecting a transnational consensus on development priorities.

Domestically, with the deepening of cultural-tourism integration and the advancement of high-quality development strategies, scholars have conducted systematic research from economic, geographic, and multidisciplinary perspectives. The construction of indicator systems for high-quality development in cultural-tourism integration has evolved from earlier reliance on single economic metrics–such as per capita GDP growth and total factor productivity–toward more diversified and comprehensive evaluation frameworks [13]. Early studies primarily assessed provincial and municipal levels of cultural-tourism integration across five dimensions: innovation, coordination, green development, openness, and shared benefits [14,15]. Subsequent research has incorporated subjective indicators–including public satisfaction with healthcare and education resources, as well as environmental quality measures such as green coverage rates and air pollution levels–thus establishing an integrated research paradigm that combines objective and subjective metrics across domains such as economic vitality, innovation efficiency, green development, quality of life, and social harmony [16].

Compared to academic theoretical frameworks, local governments’ assessments of high-quality development in cultural-tourism integration tend to prioritize performance evaluation and risk management, commonly adopting indicator systems centered on **economic transformation, innovation and reform, governance enhancement, equitable livelihood benefits, and ecological construction**. Furthermore, certain regions have introduced tailored indicators–such as **openness to foreign investment, achievements in Party-building, and urban-rural balance**–to reflect their specific resource endowments [17], highlighting the divergence between theoretical paradigms and practical application scenarios.

Current research and practice on high-quality development in cultural-tourism integration have yielded substantial results, however, several limitations remain:

1) Existing studies often fail to account for regional disparities in development stages and resource endowments, making it difficult to align with the practical needs of different areas.
2) The construction of indicator systems frequently conflates process-oriented and outcome-oriented metrics, resulting in unclear evaluation logic and a lack of focus.
3) While some scholars have extended their research to the county level, exploring the conceptual implications of high-quality development at this scale and proposing corresponding indicator systems, empirical case studies remain scarce, indicating a persistent gap between theory and practice [18].
4) Influenced by regional specificities, the high-quality development indicator systems developed by academia often diverge from the performance evaluation frameworks employed by local governments, hampering the establishment of effective synergy [19].

In light of these gaps, this study is grounded in the national strategy for high-quality development and deeply informed by local practices and regional characteristics of revolutionary base areas. Employing grounded theory method, it constructs a tailored evaluation indicator system for high-quality development in county-level cultural-tourism integration and conducts a systematic comprehensive assessment at the county scale. The research aims to address existing theoretical and practical bottlenecks, offering targeted policy insights for the revitalization of revolutionary base areas and promoting the scientific implementation and innovative application of high-quality development concepts in regions with distinct characteristics.

The academic contributions of this study are fourfold:

1) **Contextual Adaptation of Grounded Theory.** While classical Western grounded theory originated from hospital-based interviews primarily analyzing colloquial transcripts, China presents a uniquely rich and diverse landscape of textual forms (e.g., policy documents, government work reports). The innovation in applying grounded theory to Chinese materials lies not merely in “conducting research in Chinese,” but in the dynamic interaction and integration between China’s distinct cultural context, social structures, textual forms, and the methodological framework of grounded theory.
2) **Innovation in Indicator System Construction.** This study proposes a novel approach for constructing an evaluation indicator system for high-quality development in cultural-tourism integration. Moving beyond conventional indicator frameworks, it deeply integrates national strategic orientations with local development characteristics by introducing indicators such as “ **urban-rural coordination**,” “ **environmental regulation**,” “ **policy support**,” and “ **marketing**.” This dual focus ensures both the universality of the system and its specific relevance to revolutionary base areas. The inclusion of the specialized indicator “ **cultural utilization**” focuses on revitalizing red cultural resources, addressing a research gap concerning the synergy between cultural preservation and industrial development in revolutionary regions and thereby enriching the theoretical underpinnings of high-quality cultural-tourism integration.
3) **Methodological Advances in Indicator Measurement.** The study achieves methodological innovation in indicator measurement by integrating ecological and economic theories and incorporating data from new media platforms and OTA (Online Travel Agency) supply/sales metrics. Specifically, it develops **Online Public Opinion Heat Index** based on internet platform data, and constructs measurement models for **Scenic-Spot Digital Marketing Intensity Index** and **Scenic-Spot Consumption Pull Index** using OTA data. These advances enable precise monitoring of cultural-tourism market dynamics and offer new quantitative approaches for studying marketing indicators in integrated development.
4) **Practical Relevance and Policy Implications.** The research outcomes are closely aligned with the developmental realities of revolutionary base areas, clearly affirming the central role of red culture in cultural-tourism integration. The empirically validated indicator system provides a scientific basis for local governments to formulate cultural-tourism policies and optimize resource allocation. This supports the transformation of red cultural resources into economic advantages in revolutionary base areas, thereby advancing regional high-quality development.

### Conceptual definition

The term “ **integration**” originated in physics, referring to the process by which distinct substances merge into a unified whole under high-temperature melting. Later introduced into management and economics, it gave rise to concepts such as “technological convergence” and “industrial integration.” In tourism studies, integration emphasizes breaking down industrial boundaries through the penetration of elements, extended interaction, and cross-boundary sharing, thereby enabling multidimensional synergistic development and sustainable regional coherence [20].

As a development paradigm with Chinese characteristics, **cultural-tourism integration** manifests in practice through creating new business models via consumption linkages, expanding markets through resource sharing, and achieving synergistic connectivity across industrial value chains through coordinated operations and collaborative governance. Its essence lies in fostering intersections of cooperation and competitive permeation among service products, technological functions, and industrial markets, thus constructing a logically coherent and spatially relevant symbiotic system with sustainable momentum [21].

**Red culture**, rooted in China’s revolutionary history, centers on revolutionary spirit and value systems. It exists in both tangible and intangible forms, serving as a carrier of historical memory while performing functions of spiritual guidance and social empowerment in contemporary practice. It constitutes a vital source of socialist culture with Chinese characteristics. This encompasses tangible revolutionary relics such as memorial halls and martyr cemeteries, as well as intangible elements such as revolutionary ethos, traditions, and value systems [22,23].

**Revolutionary base areas** refer to regions where Communist Party organizations or revolutionary armed forces were established during the Agrarian Revolutionary War (1927–1937), the War of Resistance Against Japan (1937–1945), and the Chinese Civil War (1945–1949) under the leadership of the Communist Party of China, and where revolutionary activities were conducted over an extended period. These areas served as both the cradle and stronghold of China’s revolution, having made profound sacrifices and contributions to its ultimate victory. Most are located in remote, interprovincial border zones with relatively lagging economic development, making them key targets for national support. The *Revitalization Plan for Revolutionary Base Areas* represents a major national-level strategy in this regard.

In economic discourse, “quality” denotes the use value of a product in meeting practical needs, while “high-quality development” transcends the instrumental rationality of mere growth rate pursuit. Rather, it emphasizes a human-centered value orientation, aiming to construct an economic development model that genuinely aligns with the needs of the people [24]. Guided by the five development principles–innovation, coordination, green development, openness, and sharing-this model evaluates development levels, quality, and sustainability across multiple dimensions and layers. It specifically examines the resilience of the culture-tourism system under external shocks, the endogenous supply capacity driven by demand, the innovative vitality of industrial development, and the equitable distribution of developmental outcomes.

Accordingly, **high-quality development of cultural-tourism integration in revolutionary base areas** can be defined as: A development paradigm that leverages red culture as the core resource and empowers socio-economic progress through cross-sector integration within the tourism industry. Its connotation encompasses synergy in integrated development, alignment between tourism supply and demand, excellence in industrial performance, progress in social culture, and harmony in green development. It serves as a crucial benchmark for assessing the comprehensive revitalization of revolutionary base areas.

## Methodology

### Principles and Technical Pathway for Constructing the Evaluation Indicator System

Focused on the high-quality development of cultural-tourism integration in revolutionary base areas, this study establishes five core construction principles:

1) **National Strategic Guidance.** Aligning with national development strategies to activate endogenous regional dynamics and bridge top-level design with local implementation. Through policy direction and institutional safeguards, this principle seeks to break down geographical and administrative barriers, refine benefit-compensation mechanisms, and provide robust support for the integrated development of culture and tourism [25].
2) **Cultural Heritage and Innovation.** Centered on the core values of red culture, this principle positions the revitalization of red resources as a key pathway for deepening cultural-tourism integration. It promotes innovation through heritage preservation, facilitating the creative transformation and innovative development of red culture [26].
3) **Industrial Synergistic Advancement.** Leveraging industrial-chain agglomeration effects to drive coordinated progress across upstream and downstream sectors. This principle strengthens industrial linkages and enhances the overall competitiveness of regional cultural- tourism industries [27].
4) **Market Demand Orientation.** Driven by market needs, this principle encourages participation from diverse market actors, increases infrastructure investment, and optimizes destination-attraction strategies. By adhering to market rules, it narrows industrial-gradient disparities and enhances isomorphic benefits as well as collaborative development capacity [28].
5) **Regional Coordination and Sustainability.** Emphasizing cross-regional resource sharing and risk sharing, this principle applies green- development concepts to balance economic, social, and ecological benefits, thereby ensuring the sustainable growth of cultural- tourism industries [29].

**Fig 1:**
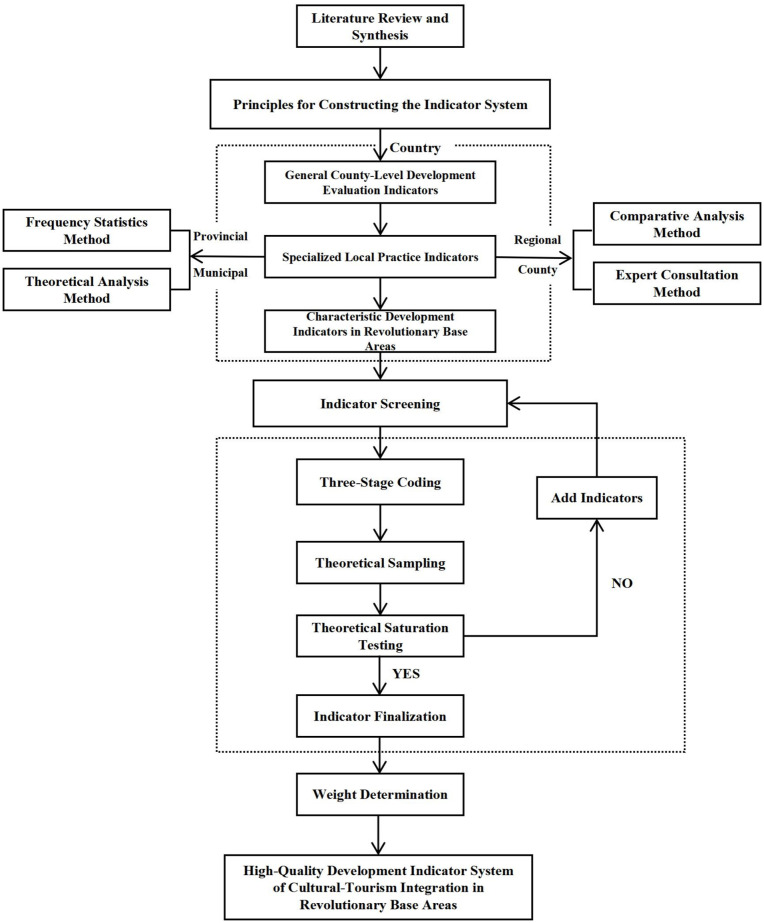
Technical pathway for cultural-tourism integration indicator system.

This study comprehensively applies grounded theory and expert-consultation methods to construct an evaluation system for high-quality development of cultural- tourism integration in revolutionary base areas. The technical pathway consists of four steps (Fig 1):

1) **Preliminary Screening of Basic Indicators.** Policy-oriented indicators were extracted from relevant policy documents, covering universal cultural-tourism categories, specific characteristics of revolutionary base areas, and distinctive regional features, yielding 563 indicators. An additional 109 indicators were identified and selected from 674 research papers.
2) **Grounded Theory-Based Indicator Consolidation.** The 563 policy-oriented and 109 literature-based indicators were merged into a preliminary indicator database. Keywords, concepts, events, and contexts within the data were identified and transformed into initial concepts. Through open coding, axial coding, selective coding, theoretical sampling, and theoretical saturation testing, an initial indicator system for high-quality development of cultural-tourism integration in revolutionary base areas was derived.
3) **Expert Consultation-Based Refinement.** The expert-consultation method was employed to revise and adjust the initial indicator system, resulting in an improved indicator system for high-quality development of cultural-tourism integration in revolutionary base areas. Each representative indicator was explained in detail.
4) **Weight Assignment.** The CRITIC weight method and an improved entropy method were used to assign comprehensive weights to the indicator system for high-quality development of cultural-tourism integration in revolutionary base areas.

### Study area

The Former Central Soviet Area of Jiangxi-Fujian-Guangdong, located in southeastern mainland China, serves as a prototypical revolutionary base area with profound historical significance. As the largest revolutionary base and the core of the Soviet movement during the Agrarian Revolutionary War, this region preserves a wealth of red cultural heritage, including: 1,088 classic red tourism sites (e.g., Jinggangshan and the Cradle of the Republic), 2,096 registered revolutionary cultural relics, 1,297 national, provincial, and municipal-level patriotic education bases.

However, constrained by geographic location, resource endowment, industrial structure, and transportation infrastructure, the Former Central Soviet Area experienced prolonged developmental stagnation. After 1949, particularly following the reform and opening-up period, its economic growth consistently lagged behind the national average: 21 of the 25 national-level poverty-stricken counties (prior to nationwide poverty eradication) were located here. Pronounced regional disparities persisted between northern Guangdong, southern Jiangxi, and western Fujian. Following *the 2012 State Council’s Opinions on Supporting the Revitalization of the Former Central Soviet Area Including Southern Jiangxi*, policy dividends catalyzed development: the tertiary sector grew by 121.42% since 2012, and per capita GDP doubled. Nevertheless, comparative analysis reveals persistent gaps in economic aggregate and regional coordinated development relative to the national county-level economy [56].

Given the distinctive historical-cultural characteristics and typical developmental challenges of the Former Central Soviet Area, its selection as the research subject carries significant exemplary value for exploring revitalization pathways in revolutionary base regions. Leveraging its advantages in red cultural resources to promote deep integration of cultural and tourism industries has become crucial for achieving high-quality development, consolidating poverty alleviation achievements, and aligning with rural revitalization strategies–representing key solutions to regional development dilemmas.

This study draws on *the Revitalization and Development Plan for the Former Central Soviet Area of Jiangxi-Fujian-Guangdong* issued by the National Development and Reform Commission in 2014. The research scope covers 108 county-level administrative units across three provinces: Jiangxi, Fujian, and Guangdong. The scope of this version of the planning area includes the entire jurisdictions of Ganzhou, Ji’an, and Xinyu in Jiangxi Province; Lichuan County, Guangchang County, Lean County, Yihuang County, Chongren County, Nanfeng County, Nancheng County, Zixi County, Jinxi County of Fuzhou; Guangfeng County, Yanshan County, Shangrao County, Hengfeng County, and Yiyang County of Shangrao; Yuanzhou District and Zhangshu City of Yichun; Anyuan District, Lianhua County, and Luxi County of Pingxiang; Yujiang District and Guixi City of Yingtan. In Fujian Province, the scope includes the entire jurisdictions of Longyan, Sanming, and Nanping; Xiangcheng District, Longhai City, Nanjing County, Pinghe County, Zhao’an County, Hua’an County, Yunxiao County, and Zhangpu County of Zhangzhou; Anxi County, Nan’an City, Yongchun County, and Dehua County of Quanzhou. In Guangdong Province, the entire jurisdiction of Meizhou is included, as well as Longchuan County, Heping County, and Lianping County of Heyuan; Raoping County of Chaozhou; and Nanxiong City of Shaoguan—covering a total of 108 counties (cities and districts).

### Data sources

Socioeconomic data were primarily obtained from the Statistical Yearbooks and Statistical Bulletins on National Economic and Social Development of Jiangxi, Fujian, and Guangdong provinces, covering 17 prefecture-level administrative units and 108 county-level administrative units within the Former Central Soviet Area. Additional data were sourced from government work reports, local scenic- area documentation, and tourism-related news releases.

Data for the **Scenic-Spot Digital Marketing Intensity Index** and the **Scenic-Spot Consumption Pull Index** were extracted from seven leading OTA (Online Travel Agency) platforms: Ctrip, Qunar, Mafengwo, Tuniu, Fliggy, Lvmama, and LY.com. The **Online Public Opinion Heat Index** was compiled from bulk data collected via Sina, Weibo, WeChat, Douyin (TikTok), and Baidu search engines.

## Results and analysis

### Preliminary indicator screening

This study constructs an evaluation indicator system for the high-quality development of cultural-tourism integration at the county level in revolutionary base areas. Adopting a three-dimensional screening logic of “ **policy orientation-theoretical support-regional adaptation**,” the design ensures the system’s authority, scientific rigor, and practical relevance. The screening process comprises two main stages:

#### Stage 1: Multi-source policy and theoretical indicator integration.

Guided by policy strategies from the Ministry of Culture and Tourism as well as provincial/regional cultural and tourism departments, this study conducted an in-depth analysis of key documents, including the *Guidelines for the Establishment of Cultural- Tourism Integration Demonstration Zones* and the *Comprehensive Tourism County Cultural-Tourism Integration Development Index Report*, to extract policy-oriented indicators. Concurrently, it systematically integrated universal county-level evaluation indicators from reports such as the *China Cultural and Tourism Integration Development Index Report (2021)* and the *China County Tourism Competitiveness Report (2020)*, together with national, regional, and provincial high-quality development index systems.

An initial pool of **511 candidate indicators** for high-quality cultural-tourism integration was compiled. Focusing on the development needs of counties in the Former Central Soviet Area and referencing post-2021 national and local policy documents (e.g., Opinions on Supporting the Revitalization and Development of Revolutionary Base Areas in the New Era), **52 regionally adaptive indicators** were further selected. These were combined with the universal indicators to form a comprehensive database of **563 indicators**, categorized into: general cultural-tourism indicators, special indicators for revolutionary base areas, and region-specific indicators. (For detailed policy and theoretical references, see Table 1.)

**Table 1:** Foundational interpretation of policy documents related to high-quality development of cultural-tourism integration.

Policy Category	Policy Name	Year Issued	Evaluation Indicators
Cultural and Tourism Development (General Category)	*Guiding Opinions on Promoting Cultural and Tourism Development*	2009	Cultural system reform, cultural industry development, tourism industry upgrading, cultural heritage protection, social harmony
	*Several Opinions on Promoting the Integrated Development of Cultural Creativity and Design Services with Related Industries*	2015	Cultural heritage protection and utilization, red tourism, characteristic cultural tourism, smart tourism
	*The 14th Five-Year Plan for Cultural and Tourism Development*	2021	Institutional mechanisms, cultural protection and utilization, industrial structure, cultural-tourism revenue, tourist visits, market order
	*The 14th Five-Year Plan for Tourism Development*	2021	Innovation-driven development, optimized layout, resource protection and utilization, product supply, consumption upgrade and expansion, market supervision, open cooperation, policy and talent support
	*Several Measures to Release Tourism Consumption Potential and Promote High-Quality Development of Tourism*	2023	Red tourism, red resources, format innovation
	*Notice on Carrying out the Construction of National Integrated Cultural and Tourism Industry Development Demonstration Zones*	2023	Coordination mechanisms, resource allocation, format innovation, policy environment, financial support
	*Domestic Tourism Enhancement Plan (2023–2025)*	2023	Promotion, tourism supply, consumption experience, public services, supply entities, market services
	*Guiding Opinions on Promoting High-Quality Development of Tourism Public Services*	2024	Public service efficiency, tourist satisfaction
Revolutionary base areas (Special Category)	*Opinions on Supporting the Revitalization and Development of revolutionary base areas in the New Era*	2021	Red culture inheritance, urban-rural coordination, infrastructure, public services, ecological civilization, industrial cooperation platforms
	*14th Five-Year Plan for the Revitalization and Development of Special-Type Regions*	2021	Policy support, key regional construction, infrastructure, green development, rural revitalization, positive urban-rural interaction
	*Implementation Plan for Supporting revolutionary base areas in Consolidating and Expanding Poverty Alleviation Achievements and Advancing Rural Revitalization During the 14th Five-Year Plan*	2021	Red heritage site protection, infrastructure, format innovation, employment expansion, promotion
	*Work Plan for Paired Cooperation Between Key Cities in revolutionary base areas*	2022	Red culture inheritance, urban-rural coordination, infrastructure, public services, ecological environment, industrial cooperation platforms
	*Key Tasks for the Revitalization and Development of revolutionary base areas in 2023*	2023	Rural revitalization, infrastructure, red culture inheritance, green development, key regions, policy support
Special Regions (Regional Category)	*Work Plan for Central State Organs and Relevant Units to Provide Pairing Assistance to the Former Central Soviet Areas Such as Southern Jiangxi in the New Era*	2021	Talent training, business environment, industrial innovation platforms
	*Fujian Province’s 14th Five-Year Special Plan for the Revitalization and Development of Old Revolutionary Areas and Soviet Zones*	2021	Economic development potential, red culture influence, livelihood welfare security, ecological environment attractiveness
	*High-Quality Development Demonstration Zone for the Ganzhou Revolutionary Old Area*	2021	Red culture inheritance, ecological environment, characteristic industry prosperity, improvement of people’s livelihoods
	*High-Quality Development Demonstration Zone for the Western Fujian Revolutionary Old Area*	2021	Ecological civilization, reform and innovation, urban-rural coordination, red culture inheritance, social livelihoods
	*Jiangxi Provincial People’s Government Implementation Opinions on Further Promoting the Revitalization and Development of Jiangxi revolutionary base areas in the New Era*	2022	Urban-rural coordination, innovation-driven development, green ecology, people’s welfare, red gene inheritance, policy guarantee

#### Stage 2: Bibliometric analysis and indicator optimization.

Based on a knowledge mapping of domestic research, a time window of **January 1, 2016–December 31, 2023** was set, with “ **culture**,” “ **tourism**,” and “ **integration**” as core keywords. A total of **674 relevant papers** were screened from CNKI, Web of Science, and other databases. Through meticulous review and in light of policy requirements and the actual development context of the Former Central Soviet Area, indicators from the literature were traced, analyzed, and categorized. After systematic refinement–including deduplication and merging of similar items– **229 indicators** were initially extracted. Further optimization yielded a final set of **109 key indicators** for the evaluation system.

Domestic scholars have constructed quantitative evaluation indicator systems from multiple perspectives to assess the quality of integrated cultural-tourism development. Relevant studies can be classified into four main perspectives: development efficiency, integration synergy, competitiveness, and high-quality development. From the **efficiency perspective**, research focuses on resource allocation and utilization, employing input-output methods to build evaluation systems that directly quantify the efficiency and benefits of integrated cultural-tourism development. The **integration synergy perspective** primarily uses coupling-coordination models to estimate the coordinated development trends between the cultural and tourism industries. From the **competitiveness perspective**, studies emphasize improvements in cultural-tourism service quality and the exploration of development potential. By establishing comprehensive evaluation indicator systems, they analyze in depth the overall strength and synergistic characteristics of the cultural-tourism industry in market competition. The **high-quality development perspective** places greater emphasis on shifts in development modes, structural optimization, and dynamic transitions within industrial integration. (Selected representative literature examples are presented in Table 2.)

**Table 2:** Foundational interpretation of literature on quality evaluation of cultural-tourism integration development.

Research Perspective	Research Content	Evaluation Indicators
Development Efficiency	Evaluation of Urban Cultural-Tourism Industry Efficiency (Wu Lihui et al., 2017) [30]	Investment amount, number of attractions, tourist numbers, revenue
	Measurement of Cultural-Tourism Integration Level and Development Trends (Wang Xiuwei, 2020) [31]	Input factors, output performance
	Measurement of Integration Efficiency of Cultural and Tourism Industries (Chen Hongling et al., 2021) [32]	Number of institutions, employees, visitor numbers, cultural business revenue, tourism revenue, tourist visits
	Analysis of Integration Trends in Cultural and Tourism Industries (Liu Honglan et al., 2023) [33]	Resources, industrial factors, labor force, output benefits
Integration Synergy	Coupling and Coordination Between Cultural Resources and Tourism Development (Sun Jianfeng et al., 2019) [34]	Cultural resource abundance, high-quality cultural resources, tourism development scale, development efficiency, development support
	Coordination Between Cultural and Tourism Industries (Liu Anle et al., 2020) [35]	Industrial foundation, human capital, industrial effects
	Development Level of Integrated Cultural-Tourism Industries (Yin Weihua et al., 2022) [36]	Comprehensive strength, operating income, institutions and employees
	Integration Level of Cultural and Tourism Industries (Wang Zhaofeng et al., 2023) [37]	Industrial foundation, industrial labor force, industrial effects
Competitiveness	Cultural-Tourism Competitiveness Evaluation (Hou Bing et al., 2016) [38]	Cultural-tourism brand resources, cultural performances and creative tourism, urban tourism market revenue
	Evaluation and Analysis of Cultural-Tourism Industry Competitiveness (Zhang Chunxiang, 2018) [39]	Resources, infrastructure, human resources, capital, knowledge
	Measurement of Cultural-Tourism Integration Potential (Xu Chunxiao et al., 2018) [40]	Resources, materials, labor force, technology
	Spatial Econometric Analysis of Cultural-Tourism Development Quality (Shi et al., 2021) [41]	Tourism revenue, employment drive, cultural heritage protection, tourist experience, sustainable resource utilization
High-Quality Development	Calculation of High-Quality Development Index for Cultural-Tourism Integration (Shi Yan et al., 2021) [15]	Financial investment, cultural development, tourism development
	Indicator System for High-Quality Development of Red Tourism (Zhang Xincheng et al., 2022) [14]	Growth drivers, structure, mode, form, outcomes, foundation
	Measurement of Cultural-Tourism Integration Development Quality (Liu Yingji et al., 2023) [42]	Government support, number of institutions, revenue, integration index

### Indicator analysis based on grounded theory

To construct an evaluation indicator system that aligns with the characteristics of revolutionary base areas, this study adopts a three-dimensional approach incorporating national strategy, local practices, and regional distinctiveness. Since conventional indicator systems often fail to adequately address the needs of special-type regions and existing theoretical frameworks provide insufficient explanation of multi-level indicator hierarchies, grounded theory was introduced for exploratory analysis. Grounded theory, a qualitative research method that derives theory from systematic data-driven analysis [43], has been widely applied across disciplines such as psychology, education, and sociology [44], and has demonstrated scientific rigor and innovation in the construction of evaluation indicator systems [45,46].

This study employs the **three-level coding process of grounded theory**–open coding, axial coding, and selective coding–to develop the logical hierarchy of indicators from the bottom up. **Open coding** conceptualizes and categorizes raw indicators. Through line-by-line and sentence-by-sentence analysis, core concepts and basic categories are extracted [47]. **Axial coding** builds on the foundational categories formed during open coding. Through relational analysis and logical clustering, main categories are integrated and structured, revealing underlying connections among indicators [48]. **Selective coding** further abstracts and generalizes the main categories to identify core categories, thereby constructing a comprehensive theoretical framework for the indicators and completing the transition from concrete indicators to abstract theory [49].

#### (1) Open coding.

The 563 indicators derived from policy-text analysis and the 109 indicators screened through bibliometric methods were merged into the preliminary indicator database for this study. Keywords, concepts, events, and contexts within the data were identified and transformed into initial conceptual labels, which were preliminarily defined and theoretically classified. Through conceptualization and categorization, a convergent analysis of the raw data was performed, yielding the initial open-coding results (see Table 3). Here, “WH” denotes red culture, “LY” denotes tourism development, and “DY” denotes regional characteristics.

**Table 3:** Open coding results of high-quality development indicators for cultural-tourism integration in revolutionary base areas.

Code No.	Subcategory	Frequency	Code No.	Subcategory	Frequency
WH1	Economic benefits of red culture	22	LY1	Economic benefits of tourism development	24
WH2	Market participation in red culture	10	LY2	Market participation in tourism development	12
WH3	Infrastructure capacity for red culture	15	LY3	Infrastructure capacity for tourism development	18
WH4	Public service investment in red culture	12	LY4	Public service investment in tourism development	15
WH5	Resource density of red culture	8	LY5	Resource density of tourist attractions	6
WH6	Resource abundance of red culture	6	LY6	Resource abundance of tourist attractions	4
WH7	Attractiveness of red culture resources	10	LY7	Marketing capability of tourism development	17
WH8	Openness level of red culture	6	LY8	External dependence of tourism development	14
WH9	Policy support for red culture	25	LY9	Policy support for tourism development	16
WH10	Comprehensive environmental effects of red culture	1	LY10	Comprehensive environmental effects of tourism development	3
WH11	Technological support for red culture	6	LY11	Technological support for tourism development	10
WH12	Scale of supply entities in red culture	5	LY12	Scale of supply entities in tourism development	7
WH13	Direct human resource scale in red culture	11	LY13	Direct human resource scale in tourism development	17
WH14	Departmental structure of red culture	2	LY14	Departmental structure of tourism development	3
WH15	Distinctive branding of red culture	6	DY15	Foundation for regional innovation achievement transformation	9
WH16	Innovation achievements in red culture	2	DY16	Scale of regional service facilities	13
WH17	Non-profit entities in red culture	5	DY17	Urban-rural coordination and integration	11

**Note on Data Presentation and Frequency Statistics:** During the analysis, although the frequency of certain codes was recorded, it served only as a tool to help researchers initially familiarize themselves with the data, ensure comprehensive coverage in analysis, and intuitively illustrate the breadth of the data to readers. All judgments regarding the importance of categories are ultimately based on the theoretical logic developed in analytical memos, their relationships with other categories, and their explanatory power regarding the core narrative. The raw data cited in the text were selected primarily for their typicality and explanatory value, rather than their frequency of occurrence.

#### (2) Axial coding.

Axial coding involves reorganizing the fragmented concepts and categories derived from open coding by identifying logical relationships among them, thereby forming a structured and hierarchical network. Building on the results of open coding, this study further examines the internal connections and potential relationships among sub-categories, identifying progressive categorical associations through causal linkages. A total of ten main categories were synthesized: cultural utilization, industrial performance, marketing, supporting services, policy support, opening-up, technological innovation, human capital, environmental regulation, and urban- rural coordination (see Table 4).

**Table 4:** Axial coding results of high-quality development indicators for cultural-tourism integration in revolutionary base areas.

Main category	Corresponding subcategories	Core characteristics
Cultural Utilization	Resource Abundance, Resource Density, Resource Attractiveness	Core foundation for cultural-tourism integration in revolutionary base areas, manifesting regional resource endowment characteristics.
Industrial Performance	Economic impact, market engagement, demand potential	Measures revenue generation and operational efficiency in revolutionary base areas’ cultural-tourism integration.
Marketing	Marketing capacity, destination recognition, specialty branding	Fundamental for resource consolidation in revolutionary areas’ cultural-tourism integration, boosting competitiveness through enhanced visibility.
Supporting Services	Provider scale, public service provision, reception capacity, facility infrastructure, departmental architecture	Essential for upgrading revolutionary areas’ cultural-tourism integration, breaking down physical/institutional barriers to stimulate market vitality and optimize resource flows.
Policy Support	Laws, incentives, compliance checks	The “1+N+X” policy system ensures institutional support for revolutionary base areas’ cultural-tourism integration through tailored policies and regional partnerships.
Opening-up	Openness, FDI reliance, international business climate	Drives revolutionary base areas’ cultural-tourism growth by leveraging foreign investment and adopting global standards for systemic openness.
Technological Innovation	Innovations, technological infrastructure, funding input	Essential for upgrading revolutionary regions’ cultural-tourism integration through: enhanced S&T inputs, talent circulation mechanisms, and industrial clustering with knowledge spillovers.
Human Capital	Direct/indirect employment	The core labor force for revolutionary areas’ cultural-tourism integration, where skill upgrades and training boost productivity via technology spillovers.
Environmental Regulation	Industrial emissions, urban contamination, ecosystem degradation, waste disposal	Facilitates transition of resource-exhausted revolutionary areas by: Decoupling development from environmental baseline, Reconfiguring factor inputs, Promoting circular economy practices.
Urban-Rural Coordination	Urbanization Rate, Income Disparity, Dual-structure Index	Urbanization levels, income gaps, rural-urban divide indicators | Drives post-poverty era growth in revolutionary areas by: Bridging rural revitalization with anti-poverty gains, Reducing regional disparities, Creating synergistic modernization pathways.

#### (3) Selective coding.

Selective coding integrates and refines categorical data to form a core category, around which relationships among other categories are systematically analyzed to construct a coherent theoretical model. Guided by systems theory, this study synthesizes the ten main categories based on the internal logic of factor-driven, effect-output, and environmental support, resulting in a 3E theoretical framework: “ **Development Elements - Development Effects - Development Environment**” (see Table 5). Adapted from the Technology-Organization-Environment model and tailored to the characteristics of cultural-tourism development in revolutionary base areas, this framework achieves an organic integration of theoretical abstraction and practical needs [50].

**Table 5:** Selective coding results of high-quality development indicators for cultural-tourism integration in revolutionary base areas.

Core Category	Main Category	Core Characteristics
Development Elements	Cultural Utilization	Prerequisite: Resource categorization, product development, and creative industry cultivation form the foundational inputs for high-quality integrated development, encompassing comprehensive investments in human, material, and financial capital.
	Supporting Services	
	Human Capital	
Development Effects	Industrial Performance	Core Output: Reflects the economic impact, social synergy, and environmental effects of high-quality cultural-tourism integration in revolutionary base areas.
	Urban-Rural Coordination	
	Environmental Regulation	
Development Environment	Policy Support	Contextual Framework: Creates an enabling environment for high-quality integrated development by reducing institutional friction and enhancing resource-to-product conversion potential.
	Opening-up	
	Technological Innovation	
	Marketing	

The model is derived from data, yet its validity and generalizability require further verification. Accordingly, this study undertook the operationalization of theoretical concepts-transforming analytical “categories” into observable and measurable “indicators” – thus establishing a measurable foundation for subsequent model validation and practical application. Adhering to the principles of operability and objectivity in quantitative indicator systems, and based on systematic simplification, an initial indicator system for high-quality development of cultural-tourism integration in revolutionary base areas was constructed by embedding the theoretical framework within yearbook databases and diverse quantitative indicator databases (see Table 6).

**Table 6:** Initial indicator system for high-quality development of cultural-tourism integration in revolutionary base areas.

Level-1 indicator (Core category)	Level-2 indicator (Main category)	Specific indicators (Subcategory)
Development Elements	Cultural Utilization	Number of patriotic education bases, total revolutionary museum resources, number of A-grade scenic spots, cultural heritage protection units at all levels, intangible cultural heritage items at all levels, museum collections, research-learning bases, certified red culture resources at all levels, total classic red tourism routes, aggregate tourism resources (including scenic areas, nature reserves, national forest parks, national wetland parks), tourism resource density (total area/county area)
	Supporting Services	POI density of catering facilities, density of star-rated hotels, number of licensed accommodation enterprises, road mileage (density), number of public toilets, number of travel agencies, fixed asset investment in star-rated hotels/travel agencies/scenic spots, local fiscal expenditure
	Human Capital	Employment in tertiary sector (star-rated hotels, travel agencies, scenic spots, hospitality industry), staff in public libraries/museums, employees in performing arts troupes, cultural market practitioners
Development Effects	Industrial Performance	Economic growth increment, coordinated economic growth rate, total factor productivity, operating income of cultural markets, cumulative visitor numbers to cultural facilities, operating income of performing arts troupes, revenue of key cultural service enterprises
	Urban-Rural Coordination	Urbanization rate, urban-rural income gap, urban-rural dual contrast coefficient, ratio of urban-rural transportation/communication expenditures, ratio of urban-rural pension insurance coverage, urban-rural per capita healthcare contrast coefficient
	Environmental Regulation	Waste treatment rate, carbon emission decoupling index, coordinated growth rate of energy consumption, investment in environmental pollution control, per capita cost of pollution control facilities, total collection of pollution fees/taxes, sulfide and dust treatment volume, carbon dioxide emission intensity
Development Environment	Policy Support	Regulations on tourism industry development, number of established cultural-tourism integration demonstration zones, number of policy design documents, proportion of cultural-tourism investment in total fiscal expenditure, number of financial service products providing capital support
	Opening-up	Number of inbound overnight tourists, inbound tourism revenue, value of foreign investment contracts, number of participants in international exchange activities, number of international exchange programs
	Technological Innovation	Number of R&D enterprises in cultural manufacturing, total R&D investment, number of tourism-related higher education institutions, enrollment in tourism-related higher education, number of academic forums on cultural industries
	Marketing	Tourism media index, tourism communication index, online marketing level of scenic spots, consumption attractiveness of scenic spots, comprehensive county-level online public opinion index

#### (4) Theoretical sampling.

The purpose of theoretical sampling is not to pursue statistical representativeness, but to obtain information from data sources that best reflect theoretical concepts, thereby developing and verifying the attributes of categories and their interrelationships [48]. The theoretical sampling process in this study consists of two main stages:

**Initial Sampling: Building a Core Concept Repository.** The objective is to identify data sources that provide broad coverage of the research phenomenon. Given that this study aims to construct an indicator system integrating both national strategic direction and local practical characteristics, two types of textual data were selected for initial sampling: **Policy Texts** – A systematic collection and analysis of policy documents such as plans, opinions, and implementation measures related to the revitalization of revolutionary base areas and the integrated development of culture and tourism at national and provincial levels. These texts represent the “national strategy” dimension, ensuring alignment between the indicator system and top-level policy design. **Academic Literature** – Bibliometric methods were used to systematically screen core journal articles and their indicator systems concerning the evaluation of cultural-tourism integration and the development of revolutionary base areas. These articles embody the “local practice” and “theoretical frontier” dimensions, guaranteeing the theoretical grounding and practical relevance of the indicator system. This stage produced a database of 672 original indicators, offering a rich conceptual foundation for open coding and providing a solid basis for theoretical construction.

**Directed and Discriminative Sampling: Deepening and Developing Categories.** During the three-level coding process, we continuously compared data with categories, as well as relationships between categories, to guide subsequent data sampling. This approach helped clarify category boundaries, verify their attributes, and explore their logical connections. In the axial coding phase, when relationships between main categories such as “policy environment” and “technological innovation” remained ambiguous, we directed attention to retrospectively retrieving and supplementing policies and literature on topics such as “scientific and technological innovation policies” and “digital cultural tourism.” This enriched the specific connotations and external associations of those categories. In the selective coding phase, to verify and consolidate the rationality of the core framework “Development Elements - Development Effects Development Environment,” discriminative sampling was conducted. We deliberately sought and compared cases from a few revolutionary base areas with sharply different economic structures and resource endowments, testing the explanatory power and adaptability of the theoretical framework across different contexts and ensuring its validity.

Through the iterative process of theoretical sampling and continuous comparative analysis described above, the theoretical model of this study gradually approached saturation and refinement. The final 3E framework systematically encompasses the core elements, output effects, and external environment of high-quality development in cultural-tourism integration in revolutionary base areas, achieving an elevation from data to theory.

#### (5) Theoretical saturation testing.

Theoretical saturation testing constitutes a crucial step in ensuring the completeness and scientific rigor of an indicator system. Its core logic lies in verifying whether the existing indicator system already covers all key dimensions–that is, whether adding new indicators would generate new theoretical categories [51,52]. In practice, this process involves conducting a point-by-point comparison between a reserved set of indicators and the constructed indicator system: if the meaning of any new indicator is fully encompassed within the existing core categories and does not introduce new influential factors among the main categories, the indicator system is considered theoretically saturated, and further expansion may be terminated. Otherwise, new indicators must be incorporated, and the coding-validation cycle repeated until both core and main categories stabilize, thereby ensuring the comprehensiveness and stability of the evaluation model.

In this study, low-frequency indicators outside the original indicator pool were further screened, consolidated, and recoded. Comparative analysis with the 10 secondary indicator dimensions revealed that the additional data no longer generated new concepts or categories, and the main categories displayed no significant shifts. This indicates that the current framework for evaluating high-quality development in cultural- tourism integration has reached theoretical saturation.

### Indicator finalization

While the initial 3E indicator database for high-quality cultural- tourism integration in revolutionary base areas was scientifically refined through grounded theory, practical constraints–such as limited data-collection channels and inconsistent statistical standards in these regions–necessitated further optimization using expert-consultation methods. This process balanced data accessibility with theoretical rigor through the following refinement strategies:

1) **Expanding Data Sources.** Going beyond traditional yearbook data by incorporating big data from OTA platforms to establish a county-level cultural-tourism consumption behavior database for the Former Central Soviet Area, ensuring “horizontal comparability and vertical additivity” of cross-regional data [53].
2) **Reducing Indicator Redundancy.** Consolidating tertiary-industry indicators through indexation–for instance, merging accommodation, catering, and travel agency metrics into composite indices.
3) **Adapting to Pandemic-Related Disruptions.** Converting absolute-value indicators into proportional metrics. To address data gaps in tourist visitation caused by COVID-19, grey prediction models were applied. Deviations were calibrated via historical-data regression analysis to maintain measurement validity during exceptional periods.
4) **Balancing Academic Depth and Practical Feasibility.** Developing secondary metrics (e.g., contribution indices, agglomeration levels, productivity ratios) for broadly defined indicators. Where county-level indicators proved difficult to measure, they were replaced with proxy variables featuring stronger data availability. Complex academic concepts were translated into executable statistical standards.

Through iterative expert review incorporating these adjustments, the refined evaluation indicator system for revolutionary base areas was finalized (see Table 7).

**Table 7:** Refined indicator system for high-quality development of cultural-tourism integration in revolutionary base areas.

Level-1 indicator	Level-2 indicator	Specific indicators
Development Elements	Cultural Utilization	**Red Culture Heritage Utilization Index** (Number of patriotic education bases, Total classic red tourism routes, Number of A-grade scenic spots)
	Supporting Services	**Reception Facility Completeness Index** (Road density, Number of public toilets per 10,000 people, Number of travel agencies, Licensed accommodation enterprises, Local fiscal investment)
	Human Capital	**Integrated Cultural-Tourism Workforce** (Employees in star-rated hotels/travel agencies/scenic spots/hospitality sector/public libraries/museums/performing arts troupes/cultural markets)
Development Effects	Industrial Performance	**Local Revenue Optimization Index** (Coordinated economic growth rate, Cumulative cultural facility visitors, Coordinated employment growth rate)
	Urban-Rural Coordination	**Urban-Rural Dual Coordination Index** (Urbanization rate, Urban-rural income gap)
	Environmental Regulation	**Green Development Coordination Index** (Coordinated energy consumption growth rate)
Development Environment	Policy Support	**Policy Support Intensity** (Tourism industry regulations, Number of cultural-tourism integration demonstration zones, Policy design documents)
	Opening-up	**Value of foreign investment contracts**
	Technological Innovation	**Local R&D Innovation Index** (Total R&D investment, Enrollment in tourism-related higher education)
	Marketing	**Online Public Opinion Heat Index** (Douyin/Weibo/WeChat/Baidu indices) **Scenic Spot Digital Marketing Index** (Promotional inputs across 7 major OTAs) **Tourism Consumption Stimulus Index** (Original formula based on distance decay theory and behavioral location theory)

### Explanation of characteristic indicators

#### (1) Development Elements (A).

This category comprises Cultural Utilization (A1), Supporting Services (A2), and Human Capital (A3), representing a comprehensive input of human, material, and financial resources that form the fundamental foundation for the integrated development of red culture-tourism.

**Cultural Utilization (A1):** Following national statistical standards, the quantity of revolutionary museums, study-tourism bases, protected cultural relic units, patriotic education bases, and graded red cultural resources is standardized under the **Number of Patriotic Education Bases (A1-1)**. Scoring is based on administrative level: national (5 points), provincial (4), municipal (3), county (2), and general (1). This index is combined and weighted with the **Number of Representative Red Tourism Routes (A1-2)** and **the Number of A-grade Scenic-Spots (A1-3)** to construct the **Red Culture Heritage Utilization Index**, representing the overall level of cultural utilization.

**Supporting Services (A2):** This dimension integrates **Road Density (A2-1), Number of Travel Agencies (A2-2), Number of Accommodation Enterprises (A2-3), Number of Public Toilets per** 10,000 **People (A2-4)**, and **Local Fiscal Investment (10,000 RMB) (A2-5)**. These variables are standardized and aggregated to form the **Reception Facility Completeness Index**, reflecting the capacity of infrastructure and public services to support red tourism.

**Human Capital (A3):** The **Number of Employees in the Tertiary Sector (A3-1)** includes staff from star-rated hotels, travel agencies, scenic areas, catering and accommodation enterprises, public libraries, museums, performing arts troupes, and cultural markets. These are consolidated into a composite indicator reflecting human- resource input in red cultural tourism.

#### (2) Development Effects (B).

This dimension evaluates the outcomes of integrated development, encompassing Industrial Performance (B1), Urban-Rural Coordination (B2), and Environmental Regulation (B3). It reflects the economic, environmental, and social synergistic impacts of red culture-tourism integration in revolutionary base areas.

**Industrial Performance (B1):** This subdimension includes: **Coordinated Economic Growth Rate (B1-1)** = ((current period value – previous period value) / previous period value) ×100%; **Number of Visitors to Cultural Facilities (B1-2)**, calculated by summing tourist visits to scenic areas, museums, libraries, cultural centers, performance venues, and travel agencies; **Coordinated Employment Growth Rate (B1-3)** = ((current year employmentprevious year employment) / previous year employment) ×100%. These three indicators are combined to form the **Local Revenue Optimization Index**, representing the core industrial benefits of integration.

**Urban-Rural Coordination (B2):** The **Urban-Rural Dual Coordination Index (B2-1)** is constructed by combining the urbanization rate (urban registered population/total population ×100%) and the inverse of the urban-rural income gap. This composite indicator reflects the degree of coordinated development between urban and rural areas under the influence of red cultural tourism.

**Environmental Regulation (B3):** The **Coordinated Energy Consumption growth Rate (B3-1)** is used to represent regional green-development capacity. It is calculated as a fixed-base growth rate: (B3-1) = ((current energy consumption – base-year energy consumption) / base-year energy consumption) ×100%. Given that integration quality is expected to generate positive externalities, the inverse of this value is taken to reverse directionality, resulting in a positive coordination index.

#### (3) Development Environment (C).

This dimension refers to the enabling environment that supports red culture-tourism integration. It includes Policy Support (C1), Opening-up (C2), Technological Innovation (C3), and Marketing (C4). Together, these reduce institutional friction and enhance the conversion of resources into effective products and services.

**Policy Support (C1):** The **Weighted Score of Policy Documents (C1-1)** is constructed by counting the number of policy documents containing keywords such as “culture and tourism,” “tourism,” “high- quality development,” and “services” on official government websites. Each document is scored according to the administrative level of issuance: national (4), provincial (3), municipal (2), and county (1). The total score reflects the policy emphasis on integration.

**Opening- up (C2):** The **Value of Foreign Investment Contracts (**10,000 RMB**) (C2-1)** represents the region’s level of openness and external investment attraction capacity.

**Technological Innovation (C3):** This includes **Total R&D Expenditure (C3-1) and Total Enrollment in Local Universities (C3-2)**. Together, they form the Local Innovation Index, reflecting the potential for knowledge generation and application in red cultural tourism.

**Marketing (C4):** The **Online Public Opinion Heat Index (C4-1)** is derived from cumulative search volumes and interaction data on major social media platforms (e.g., WeChat, Weibo, TikTok/Douyin, Baidu), representing public interest and online visibility. The frequency of scenic spots appearing in OTA tourism flow big data for each region is used to calculate the **Scenic-Spot Digital Marketing Intensity Index** $S_{i}$**(C4-2)**, achieving both “horizontal comparability and vertical additivity” of indicators within the study area. The formula is:


$$S_{i}=\left\{ \begin{array}{c} \ln q_{i},q_{i}\neq 0\\ 0,q_{i}=0 \end{array}\right.$$ (1)

where *i* denotes the specific scenic site within the region, *q*_*i*_ represents the total supply of routes and tickets for that scenic site on OTA platforms, and *S*_*i*_ is the scenic area online marketing intensity index (C4-2).

Based on behavioral location theory [54], prospect theory [55], and the principle of distance decay, this study incorporates both the objective geographic distance between tourist origins and destinations and the number of tourists (measured by bookings) to develop a metric of tourism consumption. The result is an original **Scenic-Spot Consumption Pull Index (***SCP***)** derived from OTA internet big data, expressed by:


$$C_{i}=\left\{ \begin{array}{cc} 0 & ,q_{i1}=q_{i2}=\cdots =q_{i31}=0\\ \ln\sum_{j=1}^{n}d_{ij}q_{ij} & ,q_{i}\neq 0 \end{array}\right.$$ (2)

In this equation: $d_{ij}$ represents the objective geographical distance between origin *j* and scenic destination *i*; $q_{ij}$ denotes the number of visitors from origin *j* to scenic area *i*, based on route-booking or ticket-sales data; *C*_*i*_ is the Scenic Consumption Pull Index for destination *i* (i.e., indicator C4-3). All variables are in absolute values. When the total sales volume of scenic area *i* is zero $\left(q_{i}=0\right)$, then *C*_*i*_ = 0. When $q_{i}\neq 0$, the value of *C*_*i*_ varies with both sales volume and the spatial distribution of tourist origins, increasing with larger visitor volumes and decreasing with greater distances. This index reflects the overall strength with which a scenic area attracts tourism consumption across spatial scales.

### Indicator weighting

Building upon the theoretical framework and indicator optimization, this study has developed a scientifically rigorous and operationally feasible evaluation system for high-quality integrated cultural-tourism development in revolutionary base areas. The system fully considers data accessibility, consistency in regional statistical standards, and the unique characteristics of the research subject. The finalized indicator system comprises 3 primary (system-level) indicators, 10 secondary (index-level) indicators, and 21 tertiary (evaluation-level) indicators, forming a hierarchical structure that ensures clear stratification and logical coherence.

During data processing, raw data were first normalized using the polar standard deviation method to eliminate dimensional effects. Subsequently, the CRITIC method was applied to calculate preliminary weights for each indicator, followed by a secondary calibration through entropy weighting. Through iterative computation and cross-validation between these two methods, comprehensive weights for indicators at all levels were ultimately determined (see Table 8).

**Table 8:** Weights of the high-quality development indicator system for cultural-tourism integration in revolutionary base areas.

System level	Index level	Evaluation level	CRITIC Weight (%)	Entropy Weight (%)	Combined Weight (%)
Development Elements (A)	Cultural Utilization (A1)	A1-1 Number of Patriotic Education Bases	14.974	16.197	15.5855
		A1-2 Number of Representative Red Tourism Routes	8.083	7.103	7.593
		A1-3 Number of A-level Tourist Attractions	9.011	12.084	10.5475
	Supporting Services (A2)	A2-1 Road Density (km/km^2^)	5.245	4.374	4.8095
		A2-2 Number of Travel Agencies	11.098	8.023	9.5605
		A2-3 Number of Lodging Enterprises	9.117	13.224	11.1705
		A2-4 Number of Public Toilets per 10,000 People	10.008	12.904	11.456
		A2-5 Local Fiscal Investment (10,000 RMB)	16.993	14.275	15.634
	Human Capital (A3)	A3-1 Number of Employees in the Tertiary Sector	15.471	11.816	13.6435
Development Effects (B)	Industrial Performance (B1)	B1-1 Synergistic Economic Growth Rate (%)	26.004	22.038	24.021
		B1-2 Number of Visitors to Cultural Facilities	18.073	19.938	19.0055
		B1-3 Synergistic Employment Growth Rate (%)	20.483	24.543	22.513
	Urban–Rural Coordination (B2)	B2-1 Urban–Rural Dual Coordination Index	25.941	21.031	23.486
	Environmental Regulation (B3)	B3-1 Synergistic Energy Consumption Growth Rate (%)	9.499	12.450	10.9745
Development Environment (C)	Policy Support (C1)	C1-1 Weighted Score of Policy Documents	16.997	10.863	13.930
	Opening-up (C2)	C2-1 Contract Value of Foreign Investment (10,000 RMB)	12.083	14.920	13.5015
	Technological Innovation (C3)	C3-1 R&D Expenditure (10,000 RMB)	9.024	11.903	10.4635
		C3-2 Total Enrollment in Local Universities	14.864	16.205	15.5345
	Marketing (C4)	C4-1 Composite Index of Online Public Sentiment	23.011	26.333	24.672
		C4-2 Online Marketing Intensity Index of Scenic Areas	12.388	15.084	13.736
		C4-3 Tourism Consumption-Driven Index	11.633	4.692	8.1625

### Empirical validation

The computed levels of high-quality development in cultural-tourism integration were classified into five categories–low, relatively low, medium, relatively high, and high–using the Natural Breaks method in ArcGIS. Annual visualization was conducted to further clarify the spatiotemporal evolution patterns of integrated development in the Former Central Soviet Area.

The visualization results reveal significant spatial heterogeneity in the development quality of cultural-tourism integration across the Former Central Soviet Area during the study period.

**Spatial Patterns: High-level regions** showed strong expansion momentum, evolving from scattered distribution in western and southeastern areas toward broader regional coverage. Relatively high-level regions formed stable clusters with evident agglomeration effects in central-western, southern, and eastern zones. **Medium-level regions** transitioned from initially contiguous distribution (concentrated in western, southern, and eastern areas) to fragmented, scattered patterns. **Relatively low-level regions** remained spatially stable, primarily concentrated in central and southwestern territories. **Low-level regions** continuously contracted, with only six counties (Zixi, Taining, Songxi, Guangze, Mingxi, and Qingliu) still classified at this level by 2022, highlighting persistent regional development disparities.

Among the 108 counties (cities, districts) in the Former Central Soviet Area, the top three in terms of high-quality development level of cultural-tourism integration are **Nan’an City, Longhai District, and Anxi County**, all located in southeastern Fujian Province. **Nan’an City** has integrated red culture into its key cultural system, highlighted red themes in its comprehensive tourism planning, promoted quality enhancement of scenic areas such as the Ye Fei Hometown Red Tourism Zone, and actively explored the “red tourism+” model. By incorporating elements such as characteristic agriculture, Minnan culture, and sports wellness, it has developed multiple provincial-level red-tourism boutique routes. **Longhai District**, as the birthplace of the first Party branch in the Zhangzhou region, leverages red resources such as the Longjiang Cultural Ecological Park and the Li Lin Memorial Hall to advance cultural- tourism integration and strengthen patriotic education functions. **Anxi County** has deeply explored resources such as the former site of the Annan Yongde Soviet Government and the Mo Ye Former Residence, systematically developed red-tourism routes and education-training bases, and promoted investment attraction through the Rural Revitalization Council while improving the tourism industry chain, achieving coordinated progress in cultural heritage and tourism development.

In contrast, low-level counties (such as **Taining County**) exhibit a “ **comprehensively lagging depression-type**” characteristic in high-quality development of cultural-tourism integration: red- tourism infrastructure is nearly absent, scenic-area accessibility is poor, and basic support capacities are severely inadequate. The red-tourism industry remains small in scale, weak in driving capacity, and contributes minimally to the local economy. Brand awareness is almost nonexistent, market recognition is insufficient, promotional efforts are lacking, and tourist attraction remains challenging.

**Fig 2:**
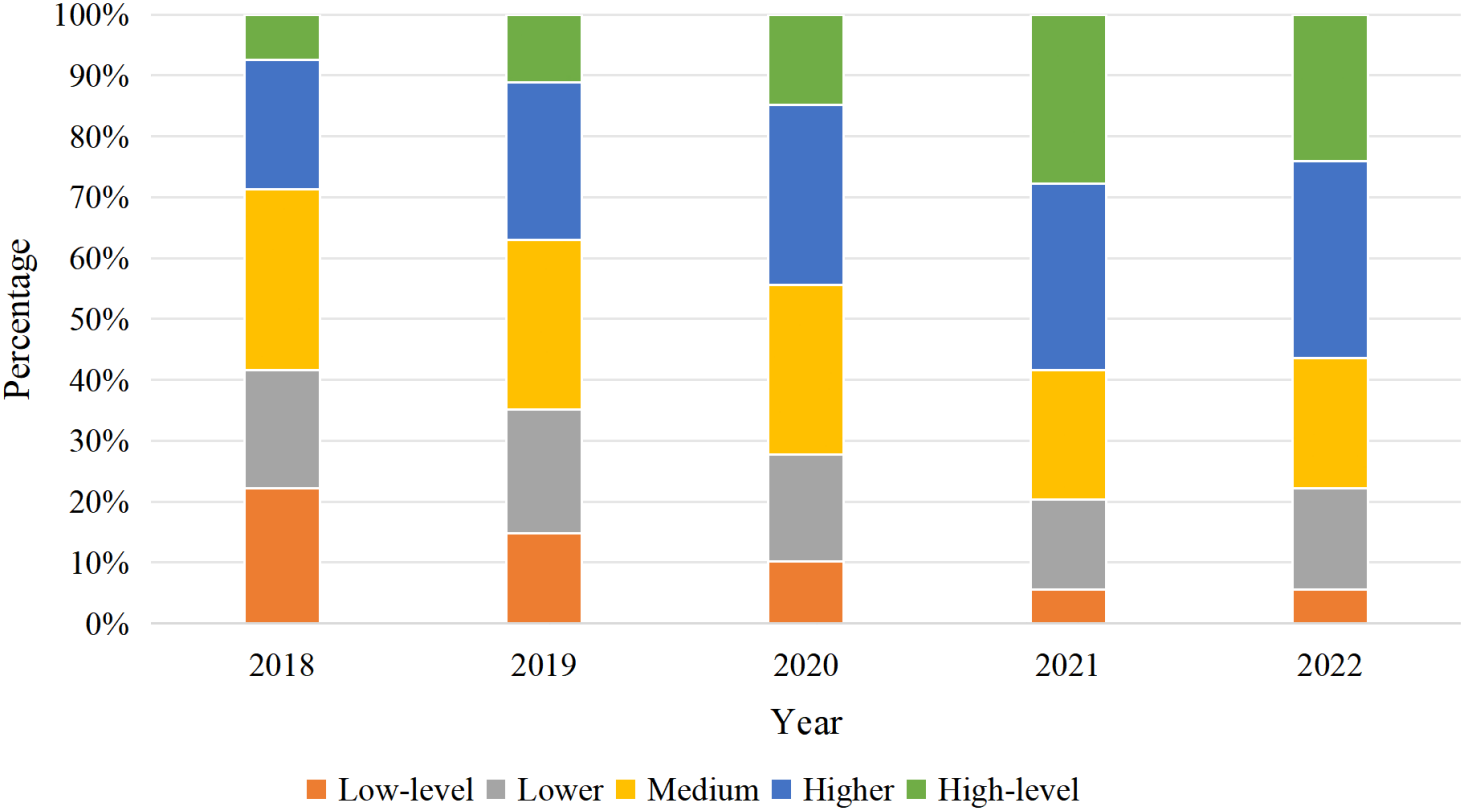
Proportions of development levels in the Former Central Soviet Area, 2018–2022.

**Quantitative Changes** (Fig 2): **Low-level regions** showed a consistent downward trend, decreasing from 24 counties in 2018–6 in 2022, representing a 75% decline. **Relatively low-level regions** first increased then decreased, starting with 21 counties in 2018, reaching a peak of 22 in 2019, and subsequently dropping to 18. **Medium-level regions** experienced continuous decline, falling from 32 counties in 2018–23 in 2022. **Relatively high-level and high-level regions** demonstrated steady growth, rising from 23 and 8 counties in 2018–35 and 26 in 2022, respectively.

To comprehensively reveal the spatial distribution characteristics of high-quality development in cultural- tourism integration within the Former Central Soviet Area, this study employs standard deviational ellipse analysis to examine the dimensions of centrality, dispersion, directionality, and spatial morphology. The standard deviational ellipse characterizes spatial structure through parameters including center, major axis, minor axis, and azimuth: the center reflects the positional centroid of element distribution, the azimuth indicates the dominant directional trend, and the major axis captures the degree of dispersion along that direction [56].

The analysis results (see Table 9) indicate that high-quality development of cultural-tourism integration in the Former Central Soviet Area generally exhibits a **northwest-southeast orientation**, primarily concentrated in the central region. During the observation period, the distribution center shifted slightly eastward and northward (longitude changed from 116.465703°E to 116.497991°E, latitude from 25.961651°N to 26.051365°N), while both the major and minor axes of the ellipse were slightly shortened. The azimuth also shifted northward by approximately 8.595°. These shifts suggest a mild spatial agglomeration trend in the quality of cultural-tourism integration development, although the overall spatial pattern remains relatively stable, with only modest fluctuations.

**Table 9:** Details of the standard deviational ellipse for the high-quality development level of cultural-tourism integration in the Former Central Soviet Area of Jiangxi-Fujian-Guangdong.

Year	Longitude of center	Latitude of center	Length of X-axis(km)	Length of Y-axis(km)	Direction angle
2018	116.465703°E	25.961651°N	2.026125	1.813424	128.002906
2019	116.47297°E	25.987674°N	2.039919	1.798759	127.939646
2020	116.488171°E	26.020433°N	2.025654	1.812473	124.157479
2021	116.489639°E	26.071061°N	2.001411	1.827436	119.226008
2022	116.497991°E	26.051365°N	2.001585	1.799499	119.408199

## Conclusions and recommendations

### Conclusions

This study responds to the practical needs of high-quality development in cultural-tourism integration within revolutionary base areas. Through systematic examination of theoretical foundations and evaluation systems, the grounded theory method was applied to construct a logical framework for a locally adapted evaluation indicator system. Using the Former Central Soviet Area of Jiangxi-Fujian- Guangdong as a case study, the applicability and scientific rigor of this indicator system were validated, offering both theoretical guidance for policy formulation and practical reference for academic research. The main conclusions are as follows:

1) High-quality development of cultural-tourism integration in revolutionary base areas is fundamentally guided by the new development philosophy, which deeply implements the principles of innovation, coordination, green development, openness, and sharing. Compared with developed coastal regions in eastern China, its developmental essence places greater emphasis on the systematic preservation and innovative inheritance of red culture, while simultaneously focusing on the synergistic advancement of economic-scale expansion and industrial-structure optimization.
2) The constructed 3E evaluation indicator system for high-quality cultural-tourism integration in revolutionary base areas comprises 3 primary (system-level) indicators, 10 secondary (index-level) indicators, and 21 tertiary (evaluation-level) indicators, each with clearly defined and interdependent functional roles. Among them, development elements serve as the foundation, providing resource, technological, and human- capital support for integration; development effects represent the objectives, directly reflecting the economic, social, and ecological outcomes of integrated development; and the development environment acts as the safeguard, including factors such as policy and market conditions, collectively forming the ecosystem for high-quality cultural-tourism integration.
3) Empirical analysis of the Former Central Soviet Area of Jiangxi-Fujian-Guangdong shows that from 2018 to 2022, the overall level of high-quality cultural-tourism integration in the region exhibited a fluctuating upward trend. Spatially, the number of low-level areas decreased from 24 to 6, while high-level areas increased from 8 to 26, demonstrating a clear pattern of contraction in low-level areas and expansion in high-level areas, accompanied by progressively intensifying spatial agglomeration.

### Recommendations

The following aspects warrant further exploration in subsequent research:

1) **Deepening Grounded Theory Methodology.** Innovative theoretical and methodological insights derived from Chinese textual materials should be further developed and communicated in frameworks and language accessible to the international academic community. This effort is not merely about sharing “China’s stories,” but also about enriching and refining grounded theory itself. By demonstrating its strong cultural adaptability and vitality, such work can help establish grounded theory as a truly global methodology in the social sciences.
2) **Addressing Data-Collection Limitations.** Although this study collected all publicly available policy documents from central and local governments, many implementation rules, internal notices, and industry-specific guidelines remained partially inaccessible or unpublished. Consequently, during later stages of analysis, it was not possible to supplement the sample with texts that could fully validate or further develop certain theoretical categories. The proposed theoretical model is therefore primarily based on macro-level policy texts. Its applicability and robustness at the micro-implementation level need to be tested and refined in future studies using more granular data, such as interviews and implementation evaluation reports.
3) **Expanding Practical Application of the Indicator System.** Follow-up research should broaden the practical application of the indicator system by conducting empirical studies in additional revolutionary base areas. Feedback from different regions should be collected to enhance the framework’s applicability and practicality, thereby offering more targeted decision-making support for high- quality cultural- tourism integration in these regions.
4) **Enhancing Big-Data Collection and Analysis.** The OTA data used in this study to construct the Scenic-Spot Consumption Pull Index may have limitations in covering specific tourist groups. Important participants in red tourism, such as elderly groups or tourists traveling in organized units (e.g., for patriotic education programs), are less likely to use online travel agency platforms for bookings. Therefore, OTA-based analyses may, to some extent, underestimate the consumption contributions and behavioral characteristics of these tourists. Future research could enhance the comprehensiveness of conclusions by integrating multi-source data-such as scenic-area ticketing system data and field surveys-for cross-validation. Furthermore, given the dynamic and complex nature of cultural-tourism development in revolutionary base areas, future research should strengthen the collection and analysis of internet-based big data. Building on the existing five-year dataset, efforts should be made to accumulate 10 -year and longer time-series data, employing spatiotemporal data-analysis models to gain deeper insights into development patterns and evolutionary trends.

## Supporting information

S1 Table Minimal data set. (XLSX)
